# Vaccination in Children with Chronic Kidney Disease: Current Status and Perspectives

**DOI:** 10.3390/vaccines14010008

**Published:** 2025-12-20

**Authors:** Maria Bitsori, Maria Michailou, Emmanouil Galanakis

**Affiliations:** 1 Department of Paediatrics, Heraklion University Hospital, 71500 Crete, Greece; med9p1110071@med.uoc.gr (M.M.);; 2 School of Medicine, University of Crete, 71003 Heraklion, Greece

**Keywords:** chronic kidney disease, vaccination, immunization, children, infection

## Abstract

Introduction: Children with chronic kidney disease (CKD) are susceptible to infections due to impaired immunity, immunosuppressive treatments, and dialysis, which lead to increased mortality, morbidity, and hospitalization rates. Immunization is an efficient preventive strategy, but despite the long-existing guidelines, vaccination rates of children with CKD remain suboptimal. Aim: This review aims to summarize the available data on vaccine-preventable infection morbidity and vaccination coverage in children with CKD, the reasons of vulnerability and suboptimal vaccination of this population and strategies that have been proposed for their overcoming. Results: Vaccination coverage studies for children with CKD are limited and outdated but, despite their variability, they confirm suboptimal vaccine coverage. The vulnerability of children with CKD to infectious dis-eases has been better understood with advanced molecular studies of their immune re-sponse. Several barriers, some of them unique to this population, hamper adherence with vaccination guidelines. Targeted interventions at different levels that have already been tried in adults with CKD, such as enhanced communication with families, cocooning strategies for the most vulnerable, education of specialists on vaccines, and organization of vaccination teams, seem promising in improving vaccination rates and infection prevention. Conclusions: The suboptimal protection from infections of children with CKD can be improved with prioritization of vaccination in their complicated care.

## 1. Introduction

Patients with chronic kidney disease (CKD) represent a growing percentage of the population, with an increasing mortality over the past thirty years [1]. The term “chronic kidney disease”, although misused as a synonym for end-stage renal disease (ESRD), is in reality a general term containing all the conditions of structural or functional anomalies of the kidneys with duration of more than 3 months [2,3]. Kidney Disease Improving Global Outcomes (KDIGO) categorizes CKD according to GFR as stages 1 to 5 and to the degree of albuminuria as stages A1 to A3 [2]. Early stage patients are almost 50 times more than ESRD patients, whose prevalence in adults is approximately 0.2% and a similar trend is noted in the limited pediatric studies [4,5,6]. The incidence of CKD in pediatric patients is estimated around 15.7–74.7 cases per million per year, which is rarer compared to adults, with regional variation due to genetic, environmental, cultural, and socioeconomic factors [5,7]. The rising tendency of the CKD population is increasingly demanding for health care systems as they require complex and expensive treatments and often present with complications, commonly infection-related [8].

CKD patients are particularly susceptible to infection due to intrinsic and environmental factors. Intrinsic susceptibility derives from the metabolic side effects of CKD, such as uremia, acidosis, and malnutrition, a state of secondary immunodeficiency associated with CKD, and immunosuppression [9,10,11]. Environmental factors include regular health care visits, exposure to resistant pathogens and interventional procedures, such as dialysis, central lines, or bladder catheterization [11]. Restrictions to antibiotic and antiviral agents hinder further the proper treatment of infections [12].

The COVID-19 pandemic highlighted the vulnerability of CKD patients to infections [13,14,15,16,17,18,19]. Infection-related morbidity and mortality rise significantly with the progressive deterioration with maximum rates of sepsis- and pneumonia-related mortality amongst adult dialysis patients [9,20,21,22,23,24,25]. Pediatric dialysis patients experience lower rates of infection-related morbidity as compared to adults, but with an increasing tendency after renal transplantation [26].

Vaccination of patients with CKD could play a crucial role in infection control. However, vaccination in this population is hampered by several factors, such as their suboptimal immune response to standard age-appropriate vaccination schedules, the need for additional doses or vaccines, and the fact that CKD patients are often managed by highly specialized health care professionals who may lack familiarity with vaccination protocols [27]. Moreover, patients themselves or their caregivers may express hesitancy toward vaccination [28]. Although there is a number of reviews on vaccination of children with CKD which state the problem of their suboptimal vaccination, few of them include data on the true burden of vaccine-preventable infections and the vaccination status of this population, which remain scattered in the existing literature. This article aims to review the existing information on vaccination status of children with CKD by focusing on infection-related morbidity and immunization rates. Also, current changes in recommendations, new vaccines, insights from the COVID-19 experience, barriers to vaccination, and interventions that could improve immunization rates in the era of vast communication possibilities are also discussed.

## 2. Methodology

This is a non-systematic narrative review that included articles found on the Pubmed search engine with the key-words “chronic kidney disease”, “ckd”, “kidney failure” and “vaccin*” or “immuniz*” and “child*” or “adolescent”, that are written in the English language without any time restrictions.

## 3. Children with CKD and Susceptibility to Infection

CKD patients present a predisposition to infections and infectious complications which is attributed to a number of factors:

*Deficient immune response:* Secondary immunodeficiency related to kidney disease (SIDKD) is a term which describes the tendency of CKD patients to infections. The lack of official criteria hinders our understanding of the extent to which it affects CKD patients, especially children for whom data is scarce [9,29,30,31,32]. The clinical manifestations of SIDKD include increased sensitivity to infections, inadequate response to vaccines, and a predisposition to cancer or autoimmune diseases. SIDKD involves both innate and adaptive immunity mechanisms, which are further affected by CKD-specific metabolic disturbances and extrinsic factors [29] (Table 1).

Innate immunity is the first line of immune defense. While neutrophil and macrophage numbers are preserved in CKD patients, disease progression is linked with defective neutrophil priming, phagocytosis, and antigen presentation. At the molecular level, the expression of Toll-like receptor 4 (TLR-4) and co-stimulatory molecule CD86, through which innate immunity cells identify foreign antigens, has been found to be decreased in CKD patients [33,34]. In addition, levels of mannose-binding lectin (MBL), an acute phase reactant protein that activates the complement cascade, were reported to differ in ESRD patients. Both, higher serum MBL, suggestive of systematic inflammation or lower levels of MBL during infections, associated with increased mortality, have been described [35]. Adaptive immune response is responsible for retainment of immunologic memory of specific antigens. In addition to the defective interaction between innate and adaptive immunity cells in CKD patients, B cell lymphopenia and imbalance between T-helper (Th)1 and Th2 lymphocytes, probably due to regulatory T cell (Treg) dysfunction, constitute a profile of suboptimal adaptive immunity [34]. Dialysis-associated hypoglobulinemia contributes further to loss of protective antibodies and increases the risk of infection [36,37].

Uremia is a main driver of the immune malfunction in CKD along with malnutrition, both sustaining oxidative stress, inflammation, endothelial dysfunction, enteral dysbiosis, metabolic derangement, and disrupted hormonal environment [34,38,39]. The inflammatory environment created by uremic toxins and its effects on innate and adaptive immunity mechanisms results in defective defense against viral infections, tumor cells, or endocytic pathogens such as tuberculosis [40,41]. Uremia has also been hypothesized to increase enteral dysbiosis [42]. The term” immunosenescence” is used to describe the changes in the immune system caused by aging, which also characterizes CKD, with molecular markers indicative of an immune system 20 years older than its calendar year, even in pediatric patients [32,43]. In adult patients, this finding is probably exacerbated by CMV latency [44,45] and cannot be reversed with kidney transplant [46].

*Endocrine consequences of CKD:* These consequences of CKD include disrupted bone metabolism characterized by hyperphosphatemia, hypocalcemia, secondary hyperparathyroidism, reduced vitamin D and growth failure, erythropoietin deficiency, renin-aldosterone system dysregulation, and changes in levels of adipokines with important effects on the secondary immunodeficiency profile [47]. In addition, the tremendous increase in the key molecule FGF23 in CKD, which is secreted by fibroblasts and regulates renal excretion of phosphate and vitamin D metabolism, negatively affects the immune system and has been linked with increased mortality [47]. Erythropoietin deficiency and resistance to exogenous administration increase the need for intravenous iron infusions. Iron therapy predisposes patients to bacterial and fungal infections [34]. The immunomodulatory properties of renin-aldosterone system on chemotaxis, cellular adhesion, and lymphocyte activation are adversely affected in CKD, as is its role in blood pressure regulation [9,48]. The disruption of the levels of adipokines and neuropeptides such as leptin, resistin, neuropeptide Y (NPY), and retinol binding protein 4 (RBP-4) have also been suggested to contribute to CKD immunodeficiency [48].

*Increased exposure to infections:* CKD patients of the later stages are frequently exposed to hospital environment and resistant pathogens and are at increased risk for invasive infections [27]. Frequent hospital visits expose these patients to crowded environments and increase the risk of transmission of airborne pathogens such as COVID-19, influenza, and tuberculosis [14,49], or infested surfaces which enhance transmission of resistant microbes such as *Staphylococcus* spp. [50], or viral agents through skin contact [51]. Skin carriage of staphylococci puts CKD patients at risk of bacteremia or peritonitis, depending on dialysis mode [52]. The vascular or peritoneal devices of ESRD patients disrupt the skin barrier and facilitate pathogen insertion directly into the blood stream or peritoneal cavity, resulting in invasive and occasionally fatal infections given the immunocompromised status of these patients [52]. In addition, the frequent use of urine catheters in CKD patients, especially in the context of limited renal function or dysfunctional anatomy, increases the risk of severe upper urinary tract infections [53]. The restrictions in antibiotic administration due to reduced renal function complicate further the patients’ course.

## 4. Vaccine-Preventable Infection in Children with CKD

Whereas all-cause and infection-related hospitalization rates are higher in children with CKD, evidence about vaccine-preventable diseases (VPD) morbidity and mortality is limited. Hepatitis B virus (HBV), *Streptococcus pneumoniae*, influenza virus, varicella-zoster virus (VZV), measles virus (MV), human papilloma virus (HPV), and recently COVID-19 infections are particularly important for these patients. VPD morbidity data of children with CKD is limited and outdated with most recent studies focusing exclusively on COVID-19 pandemic and are summarized in Table 2. Vaccination could particularly benefit CKD patients, but despite the numerous guidelines vaccination rates are lower in these patients than in the general population, even in childhood. In a European multicenter study of kidney transplant (KT) candidate children, only 8.7% of the participants were completely vaccinated according to guidelines [54]. Each of the recommended vaccines is met with distinct challenges in clinical practice. Studies of vaccine coverage in children with CKD are summarized in Table 3.

## 5. Vaccine-Preventable Infections of Particular Importance for Children with CKD

*COVID-19 infection:* COVID-19 pandemic challenged the management of children with CKD, as it hindered their accessibility to health care units, set back their chronic disease control, and increased the risk of associated complications. The most stable finding of the studies that included pediatric CKD population was an increased COVID-19 hospitalization risk of CKD children in comparison to healthy ones, with odds ratios (OR) reaching 11.4 [17,18,19,62]. CKD severity seems to be irrelevant COVID-19 morbidity. A systematic review concluded that children with nephrotic syndrome are not at increased risk for severe COVID-19, even though relapses of their disease could complicate their course [61]. A report from India, however, suggested that nephrotic children are at increased risk for complicated COVID-19 infection and so are CKD children [59,60]. All these studies indicate towards increased morbidity in children with CKD; however, timing issues, such as vaccine availability and local epidemiology, such as prevalent variant, hinder direct comparison with COVID-19 vaccine immunogenicity and safety in pediatric CKD patients has been reported only for mRNA Biontech/Pfizer vaccine BNT162b2 in two studies, both of which concluded that only immunosuppressed CKD and KT children displayed much lower levels of immunoconversion [71,72]. A further study on CKD adolescents did not validate any safety concerns [73].

*Influenza infection:* Influenza infection is associated with significant short- and long-term morbidity and mortality in extremes of age, and in comorbidities [74]. Data for morbidity, specifically of CKD children, are limited. Vaccination rates amongst CKD adults range between 28.3 and 73.4% [66,70,75,76,77,78,79,80], whereas in pediatric CKD patients, rates converge around 40% with variation between different CKD groups despite the different end-points (complete or seasonal vaccination) [55,64,69]. Despite the concerns for decreased immune response of ESRD patients to influenza vaccines, the vacinees presented with lower influenza-related hospitalization rates and mortality than those not vaccinated [81].

*Streptococcus pneumoniae infection*: Young CKD patients are particularly vulnerable to invasive pneumococcal disease (IPD) [82]. The estimated morbidity and mortality of pneumococcal meningitis in children reach 20–30% and 5–40%, respectively, but CKD patients, especially those on dialysis, exhibit a 10-fold mortality rate [83]. Vaccination coverage amongst adult CKD patients varies between 1.2 and 53% [75,78,79,80,84] and the same variability has been reported for CKD children [80,81]. Reports for young CKD patients from the US renal data system showed vaccination coverage between 10 and 20% [80], dialysis pediatric patients reached rates 32.9–70.6% in multicenter and observational studies [77,82] and highest vaccination rates of 70.6% were reported in young patients with nephrotic syndrome [85] (Table 3). Immunogenicity was approached in several studies involving CKD children from different methodological standpoints, and all of them concluded that vaccination with PPSV and PCV vaccines is safe and adequately immunogenic in CKD and nephrotic children with the exception of those under immunosuppressive therapy, like KT recipients [67,83,86,87,88]. The introduction of PCV20 vaccine in the vaccination programs of several countries poses the question of whether the substitution of pneumocococcal polysaccharide vaccine is adequately safe or immunogenic in CKD population [89]. There is a lack of studies focused on children with CKD; however, a recent study estimates that PCV20 will aid with the reduction in IPD morbidity in children with immunosuppression or other comorbidities and the financial burden for the health care system [90].

*Hepatitis B infection:* Dialysis, frequent skin breaches, and CKD-associated immunodeficiency are responsible for CKD patients’ vulnerability to HBV infection and chronic carriage, cirrhosis, and hepatocellular carcinoma [91]. Higher HbsAg positivity has been found amongst hemodialyzed adult ESRD patients in comparison to the general population, though this trend has not been confirmed for pediatric dialysis patients in a single study in Bangladesh [56]. In the same study, however, it was found that nearly 70% of the participants lack protective serologic antibody titers, and similar rate of hypo-responsiveness among children on dialysis was reported in a study from Egypt, with risk factors of suboptimal immune response being male gender, dialysis duration more than five years, and HCV seropositivity [56,91]. Our review of the literature demonstrates a lack of pediatric studies in other geographical areas with different seroprevalence of HBV. The timing of HBV immunization seems to be a key-factor for preservation of immunity, since children who were immunized during infancy were six times less likely to experience fading HBV immunity in comparison to children who were vaccinated post-dialysis [54,92,93]. Several strategies have been adopted to improve seroconversion and maintenance of protective antibody levels in CKD patients such as adjuvanted vaccines, vaccines with increased antigen concentration or additional dose regimens. The most preferred strategy for children has been the use of increased dose regimens [94,95,96]. Adjuvanted vaccines and double antigen concentration vaccines are not licensed for pediatric use. Regarding vaccination rates, it was found to be 63% in 46 KT children in a study from Brazil [68].

*Measles:* Measles continues to be a threat, despite the effective measles-mumps-rubella (MMR) vaccine and the decrease in endemic transmission in western countries [91]. Immigration from countries with incomplete or no vaccination programs, isolated communities with certain anti-vaccine beliefs, and exaggerated publicity of side effects of the MMR vaccine have increased the risk of measles outbreaks [97]. Immunocompromised patients, although at risk of severe manifestations, cannot be safely protected with immunization as MMR vaccine is contraindicated in their case [98]. Children with CKD who are not on immunosuppressive treatment seem to respond adequately to MMR with development of protective antibody titers, even while on dialysis, despite the CKD-associated defective T-cell immune response [98]. A recent case–control study from India showed that CKD children have non-inferior seroprotection for measles, with rates of 90%, similar to those of healthy children, but with a declining tendency in advanced CKD of stage 3 and beyond [99]. There are only limited reports on MMR vaccination rates of CKD children, mostly based on serosurvey data from KT candidates. A recent study from Turkey showed that only 59% of KT candidate children had protective antibody titers against measles [100]. The question of whether children can receive the MMR vaccine after KT, being on immunosuppression, or being on steroids, is addressed in recent studies, which shows that MMR vaccination could be considered for patients on minimal immunosuppression or minimal steroid dose on an individualized basis [101].

*Varicella-zoster:* Varicella infection is a major threat for immunocompromised patients, especially KT recipients with a mortality rate of 34% in a systematic review of adult CKD patients [102]. Although pediatric CKD patients show significantly lower morbidity and mortality rates, several cases of disseminated disease or even death have been described [58,103,104,105]. Preventive VZV strategies include post-exposure prophylaxis with varicella-zoster immune globulin (VZIG) and antiviral therapy; however, vaccination is the most effective one [106]. CKD children not on immunosuppressive therapy show sufficient immunoconversion after vaccination in a single-center study in the UK [107]. A significant proportion of children KT recipients who are immune to VZV before transplantation seem to retain adequate level of antibodies after grafting as 62% of 186 children retained sufficient antibody titers at one year and 42% at ten years after transplantation [101]. This study also underscored the vaccine’s efficacy as vaccinees experienced less varicella infections compared to unvaccinated patients (10% vs. 44%) [57]. According to current guidelines, live-attenuated VZV vaccine is contraindicated for children on steroids or other immunosuppressive medication. The recombinant zoster vaccine (RZV), which has recently been introduced and licensed for individuals over 18, significantly advanced the prevention of herpes zoster, especially among immunocompromised adults [108]. Its use in the immunocompromised pediatric population, although promising, has not been extensively evaluated.

*Human Papilloma Virus (HPV) infection:* HPV is an important contributor in the pathogenesis of human cancers and immune dysregulation plays a major role in the enhancement of the virus’ oncogenetic properties. Therefore, ESRD patients and KT candidates should be at the forefront of preventive measures, which include HPV vaccination [109]. Hocker et al. found that only 27.3% of 254 ESRD pediatric patients were vaccinated for HPV [54], despite the safety and efficacy of the vaccine even for KT recipients [109,110].

### Other Vaccine-Preventable Infections and Data for CKD Children

*Diphtheria-tetanus-tertussis:* DTaP vaccine is universally included in vaccination programs and vaccination rates are often used to evaluate the successful implementation of a country’s vaccination program [111]. However, there are no studies on the vaccination coverage of children with CKD. HD adult patients are reported to have a vaccination coverage of approximately 24% [112]. Studies of pediatric KT recipients also showed seroprotection, with protective titers in only 57.4% of patients for diphtheria and 81.5% for tetanus [113,114]. Children with nephrotic syndrome seem to have lower protective titers as well, especially those with steroid-resistant disease [115].

*Meningococcal infections:* CKD patients are not traditionally considered particularly susceptible to meningococcal disease unless there is an underlying comorbidity or immunosuppressive therapy with complement inhibitors. The growing use of complement inhibitors in diseases such as atypical hemolytic uremic syndrome (aHUS) raises questions about the efficacy of meningococcal vaccines in CKD patients with aHUS [116]. Literature addressing this question is limited, but there are concerns that immunological response is probably suboptimal. Regarding vaccination coverage, it remains low, with only 47.9% of pediatric KT candidates being fully vaccinated against meningococcal infections [54].

*Tuberculosis:* CKD patients have a higher tuberculosis prevalence than the general population, with most affected groups being ESRD and KT patients, probably due to SIDKD, comorbidities, immunosuppression, or socioeconomic conditions [117,118]. Adult HD patients have a 10–25-fold increase in relative risk of symptomatic tuberculosis and in KT recipients the risk is even higher [49]. The frequency of tuberculosis among pediatric KT patients varies depending on the study and the local epidemiology, from 0.86% in 345 patients in Spain [118] to 9.7% of 72 patients in South Africa [119]. CKD and transplantation are not universal indications for BCG vaccination but screening for TB prior to administration of immunosuppressive therapy or initiation of dialysis is highly recommended [117].

## 6. Barriers to Vaccination

The importance of vaccination in CKD patients is well-established by numerous guidelines. However, vaccination rates in CKD children remain far from ideal. A variety of barriers in the general population, such as socioeconomic status, accessibility to health care services, or personal and philosophical attitudes towards immunization, are further exaggerated in the CKD population with safety issues and complexity of medical management of their condition (Table 5).

*Unfamiliarity with the guidelines:* Children with CKD are subjected to modifications in their vaccine schedules, which causes confusion to both parents and health care professionals. Tran et al. showed that pediatric nephrologists often do not follow ACIP guidelines for live vaccines [120]. Adequate knowledge has been reported in only 22% of health care providers, with the most usual deviation being the refusal to administrate vaccines to patients on low dose steroids [111]. Similarly, the vast majority of parents of children with CKD are not aware of the recent vaccination guidelines, with only 1% being familiar with live vaccine indications [120].

*Confusion of responsibilities:* Vaccination in children with CKD, who are looked after by highly specialized physicians, is overlooked because neither the specialists nor community pediatricians take over their immunization responsibility. Community physicians are often unaware of the most recent guidelines or hesitate to vaccinate this patient group due to disease severity, immunosuppressive therapy or fear of relapses. One of the most understated drivers of neglect is the simple oversight of the health care system to clearly delegate vaccination supervision and implementation for these children [55,64,121]. Printza et al., in their study on promoters of H1N1 vaccination of children with CKD, at the height of H1N1 pandemic, highlighted the importance of recommendation by pediatric nephrologists [79]. Parents’ higher educational level and children’s severity of renal disease positively affected vaccination rates [64]. Similarly, Scheuerman et al. reported that 38% of the parents of non-vaccinated against influenza children would have chosen otherwise, had they been informed by their nephrologist [55].

*Live vaccine safety concerns*: Major safety issues have been raised in the vaccination of immunocompromised individuals with live vaccines [102,122]. The risk depends on the degree of immunosuppression and the underlying disease. In children with CKD, MMR is considered safe, unless they are on immunosuppressive therapy, either with Disease Modifying Antirheumatic Drugs (DMARDs) or with biological agents [123]. Even then, experts can decide to vaccinate these children [124]. Kamei et al., in their study of 60 nephrotic children on immunosuppressive agents, administered MMR and VZV vaccines in cases fulfilling certain immunological criteria, such as normal cellular immunity, disease on remission for over 6 months, and steroid therapy < 1 mg/kg/day [125,126]. These children had sufficient immunogenicity and no breakthrough infections or serious side effects. This study highlights the safety and efficacy of live vaccines in children with intermediate levels of immunosuppression, but further research is warranted [125,126]. Despite this encouraging research, physicians remain reluctant to decide vaccination, and the parents cannot be easily convinced of the need and safety of live vaccines in the case of their children [127,128].

*Vaccine hesitancy:* Vaccine hesitancy describes the patients’/parents’ tendency to delay or deny vaccines despite availability, ranging from refusal or doubt for certain vaccines to complete denial of all vaccinations. In adult patients, uptake of COVID-19 vaccine was found to be negatively associated with social deprivation, minorities, and mental illness, while immunosuppression as well as trust and good communication with health care professionals were positively associated [129,130]. In the case of children with CKD, the choice to vaccinate is under parents’ responsibility, who are often more hesitant to vaccinate their child than they are themselves. Wang et al., in their study of 207 children with CKD, found that more than 2/3 of the parents expressed uncertainty to vaccinate their child againstCOVID-19, and these parents were more likely to be hesitant towards other routine childhood vaccines, including influenza [28]. Epidemiological characteristics that were associated with higher vaccination uptake were older age, female gender, and glomerulonephritis over Congenital Anomalies of the Kidney and Urinary Tract (CAKUT) [36]. Parents more willing to vaccinate their children were influenced by the “benefit vs. harm” concept and by the trust to their physician [28].

*Vaccine distribution:* Vaccine uptake, apart from hesitancy, can be influenced by vaccine shortages and distribution challenges, especially in low-income countries. COVID-19 pandemic showcased the inequalities in vaccine distribution amongst nations, which were influenced by socioeconomic or religious factors and health care system organization and accessibility [130,131]. Even in developed countries, administration of important vaccines in ESRD patients often demands systematic effort and organization from the part of the health care providers.

## 7. Recommendations: General Principles and Specific Aspects

SIDKD is a major contributor to the vulnerability of CKD patients to vaccine-preventable infections and its consequences are proportional to the progression of CKD [29]. The lack of a precise definition makes epidemiological data scarce, especially in children, and clinical diagnosis difficult. However, the general biomarkers of immunodeficiency, such as white blood cell count, total Ig levels, measurement of antibodies to specific pathogens or vaccine responses, complement factors, and immunophenotyping, might be useful for the evaluation of CKD children as the disease progresses [29]

Immunization guidelines for children with CKD have existed for long and vary between countries and health care authorities. This variation, together with the different ages and disease status, are often a source of confusion. Nevertheless, vaccination approaches for children with CKD can be easily perceived on the basis of the following general principles [27,122,128,132]:

- Vaccination can be performed according to the national vaccination program except for immunocompromised CKD or KT children, in whom live vaccine administration is contraindicated.
- Stage 4 and 5 pre-dialysis children might need supplementary vaccination, such as additional pneumococcal vaccine or additional doses of HBV vaccine. Earlier administration induces better immune response and more sufficient protection. Accordingly, vaccination status for *S. pneumoniae* should be reviewed and annual check of anti-HBs antibodies should be scheduled.
- KT candidates should complete vaccination, ideally 4 weeks prior to transplantation for live vaccines and 2 weeks for inactivated vaccines.
- Children with nephrotic syndrome are eligible for supplemental PCV vaccination.
- Screening for tuberculosis should be performed pre-dialysis, pre-transplantation, or before the initiation of immunosuppressive therapy
- Pre-transplantation serological titers for HBV, HCV, HIV, EBV, and CMV should be considered.
- All CKD, KT, and nephrotic children should be vaccinated for influenza and COVID-19 annually.
- HPV vaccination is advisable in children over 11 years old who are about to initiate dialysis, immunosuppressive treatment, or are candidates for KT.

On the basis of main current guidelines, specific considerations that should also be taken into account are summarized in Table 4 [124,133,134,135].

Regarding specific immunization scheduling for children with CKD, the following subgroups should be considered:

*Nephrotic syndrome:* Children with nephrotic syndrome present a noteworthy predisposition to IPV due to the loss of immunoglobins associated with their condition. The addition of 23-valent pneumococcal polysaccharide vaccine (PPSV23) in their vaccination regime has no longer been necessary since the licensing of the newest PCV20 vaccine, a single dose of which can suffice. Special consideration should be given to a subgroup of early onset nephrotic syndrome patients who are unable to complete their vaccination with MMR and VZV due to their constant need for immunosuppression [136]. In this case the risks between a possible infection, especially in the setting of an outbreak, and the possible side effects of vaccination should be weighed. The guidance from EULAR for vaccination of children with rheumatic disorders under immunosuppressive therapy can be taken into account for nephrotic children; according to this guidance, booster MMR and VZV vaccination can be considered in patients under biological factors, such as anti-IL-1 or anti-TNF, but not on anti-CD20 or on high-dose corticosteroid therapy [124].

*Pre-dialysis patients (stage 4 and stage 5 of CKD):* This CKD stage is a preparatory one for children and families for possible initiation of dialysis and discussion of KT and a window for immunization review. Anti-HBs antibodies should be checked and, if necessary, repetitive doses administered [137]. Furthermore, an additional dose of PCV20 and HPV vaccine in age-appropriate patients should be administered [133,134,135]. These immunizations are extremely important as progression of CKD adversely affects the efficacy of vaccination. Annual influenza and COVID-19 vaccinations should not be overlooked. Moreover, due to the higher incidence of tuberculosis in hemodialysis centers, screening for TB should be performed [138].

*Pre-kidney transplant immunizations:* All age-appropriate immunizations should be completed a month before transplantation. KT patients go through severe immunosuppressive therapy and are especially vulnerable to CMV, EBV, or HPV reactivation. Therefore, CMV, EBV, HIV, HBV, HAV, and tuberculosis screening should be performed prior to KT [109]. Post-KT, these patients can restart immunizations 3–6 months after grafting for influenza, COVID-19, and *S. pneumoniae* and a year for the other non-live vaccines. Vaccination with live vaccines is contra-indicated; however, it can be considered in certain cases on minimal immunosuppression [139].

## 8. Future Perspectives and Interventions for Improvement

To improve vaccination rates in children with CKD, a multi-faceted approach is required, targeting both families and attending physicians (Table 5).

*Health care system organization:* Targeted nurse-led interventions and electronic health record (EHR) system reminders can be combined in sustainable vaccination plans for children with CKD. Hepatitis B coverage in adult CKD patients was successfully increased from 18% to 58% within a period of 6 months by use of EHR to track the candidates and nurse appointments to inform patients and administer the vaccine [140]. In a similar approach, pneumococcal vaccination coverage of 68 children with nephrotic syndrome was increased from 12% to 86% through educational intervention to health care providers [140]. Malone et al. managed to increase pneumococcal vaccination coverage in 122 KT children from 6% to 52% with qualitative interventions, such as the use of an algorithm to pick eligible patients and set up of reminders for physicians [141]. Similarly, nurse-led initiatives in hemodialysis units increased patients’ vaccination rates [142]. The incorporation of such measures in the demanding and busy daily practice of dialysis units and specialty clinics might sound only theoretical but results of the admittedly limited studies so far showed that they work in real life. The key for their implementation is the definition of standard teams, which will systematically track patients in need of vaccination in a timely manner and schedule vaccination visits. These practices could be proven significantly cost-effective in the long-term with the reduction in the financial burden of infectious-related hospitalization as limited pediatric CKD studies show [90,143].

*Targeted medical research:* Gaps in medical knowledge become apparent on reviewing the literature for evidence-based immunization recommendations for pediatric CKD population. There is a lack of research on immunogenicity and safety of vaccines particularly important for children with CKD, for instance HBV vaccination. Consequently, decision-making is often based on data derived from adults. Research data on vaccine coverage for pediatric CKD patients are mostly outdated and the subgroup of early-stage CKD patients is under-represented. Furthermore, the lack of studies focusing on enhanced HBV vaccination in pediatric patients leads to a lack of specific recommendations on vaccine choices, doses, intervals of antibody checks, and booster vaccine administration in most guidelines for the group of 0–16-year-old ESRD patients [134,135].

*Physicians’ awareness:* Physicians with knowledge of guidelines and awareness of adverse effects and safety profiles could greatly contribute to follow-up of patients’ vaccination status, more efficient planning of vaccine administration, and enhancement of doctor-patient interaction [55,64]. Children with CKD are often managed by specialized medical professionals who might not be trained for the importance of vaccine administration. As a result, they overlook vaccinations and are not efficient in answering the caregivers’ questions or addressing hesitancy due to the parents’/patients’ individual beliefs and concerns. In addition, community pediatricians are often reluctant to interfere in chronic patients, believing that their vaccination plan is also under the specialists’ responsibility [121].

*Cocooning strategy:* This term refers to protection of a vulnerable individual who cannot be effectively vaccinated due to age, immunodeficiency, or other restrictions, with a circle of immunity formed by vaccination of close contacts. An example of implementation of the cocooning strategy is parents’ immunization for pertussis to protect their infants. Cocooning attempts to utilize altruism as a motivator for immunization, which might be more successful than vague recommendations or mandatory policies [144]. In the case of children with CKD, influenza, and COVID-19, which are highly transmissible infections with adverse effects on renal function, they can be effectively prevented by vaccination of close contacts, since patient vaccination might be of decreased efficacy. Similarly, children with nephrotic syndrome or under immunosuppressive therapy for renal disease or KT who cannot receive live vaccines and are highly susceptible for severe or even fatal measles and varicella can be successfully protected by cocooning strategy [27].

## 9. Limitations

This is a narrative review that was conducted in a non-systematic manner and without a defined timeframe for searches. Furthermore, the lack of recent research focused on vaccine coverage, safety, and immunogenicity on older or even recently introduced vaccines restricts our presentation of the current vaccination status in children with CKD. Our deductions derive from observational studies from heterogenous populations, different timeframes, and socioeconomic backgrounds which were probably vaccinated according to different vaccination programs. This variability is also present in immunogenicity studies, where several methods and cut-off values differ according to the study and influence the ultimate results.

Infection prevention and vaccination are topics of increased significance for this patient group with consequences to their short- and long-term outcomes as well as impact on the economical expenditures of the health system. Proper recording of vaccination rates could assist the achievement of these goals and further scientific research and health care organization need to ensue.

## 10. Conclusions

Children with CKD are a vulnerable group and improving their vaccination is important for the reduction in vaccine-preventable diseases. Children with CKD present a significant degree of immunosuppression that affects vaccination efficacy and raises safety concerns. Vaccination rates in this group remain suboptimal for most vaccines due to hesitancy, unclear recommendations, confused physician responsibilities, vaccine availability, or vagueness of guidelines. Simple interventions, such as regular review of patients’ vaccination records by designated personnel, electronic reminders, education of physicians and families, and promoting cocooning practices, could be very successful in increasing vaccination rates and improving this patient group’s quality of life.

## Figures and Tables

**Table 1:** Causes of increased susceptibility to infection in children with CKD.

Secondary Immunodeficiency Related to Kidney Disease (SIDKD)	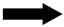	↑ Susceptibility to Infection
		↓ Response to Vaccines
		↑ Risk for Cancer/Autoimmune Disease
**Contributing factors**		
Increased exposure to infection	Frequent hospital visits Dialysis devices	
CKD/nephrotic-associated medications	Steroids Immunosuppressants Iron agents	
CKD/Dialysis Consequences	Malnutrition Anemia Hypoalbuminemia	
Uremic environment	Enteral dysbiosis Persistent inflammation Direct effects on cellular compartment of immunity	
Immunosenescence	Advanced immune decay Molecular markers of immune system 20 years older than patient’s age	
Endocrine consequences of CKD	Disrupted bone metabolism: ↓ 1,25(OH)2D and ↑ FGF23: reduced immunomodulatory action and renin-aldosterone system (RAS) suppression Disturbed RAS function: hypertension, loss of its immunomodulatory role Increased adipokines and neuropeptides	
**Distinct mechanisms**		
Innate Immunity	Defective neutrophil priming Reactive oxygen species (ROS) imbalance Defective phagocytosis Defective antigen presentation	
Adaptive Immunity	B-cell lymphopenia T-regulatory (Treg) lymphocytes dysfunction Th1/Th2 imbalance	

ROS: reactive oxygen species, Treg: T regulatory lymphocytes, Th: T helper lymphocytes, FGF: Fibroblast Growing Factor, RAS: Renin-Angiotensin System, ↑: increased, ↓: decreased.

**Table 2:** Infection-related (VPD included) morbidity/mortality of children with CKD.

Study Year /Country	Infection	Morbidity/Mortality	Population	Ref.
2007/USA	All infection-related hospitalization	39.9% HD, 51.2% PD 47.4% KT	ESRD children (n = 3580) vs. adults 1996–2001	[26]
2013/USA	All infection-related and cardiovascular mortality	<5 yrs 112.2 → 83.4/1000 py >5 yrs 44.6 → 25.9/1000 py	ESRD children (n = 23,401) mortality rates 1990–1994 vs. 2005–2010	[25]
2021/USA	All cause hospitalization	CKD prevalence among discharges: 3.9% Mortality OR: 1.51 (95% CI: 1.40–1.63)	CKD vs. non-CKD children, 2006, 2009, 2012, 2016	[8]
2008/Denmark	Invasive pneumococcal disease (IPD)	CKD prevalence among IPD cases: 0.36% Morbidity RR: 18.9% (95% CI: 2.8–127.1)	1655 IPD cases 1977–2005, chronic conditions (including CKD) vs. non-chronic conditions	[55]
2021/ Bangladesh	HBsAg positivity	No positive patients	35 CKD children during 2021	[56]
1997/USA	Varicella	66 positive patients (no calculated rates)	KT children 1984–1996	[57]
1992/USA	Varicella	Morbidity: 9.6% (n = 8) Mortality: 2.4% (n = 2)	83 KT children 1979–1991	[58]
2023/Brazil	COVID-19	Mortality: 20.8% (n = 59) Mortality non-KD: 7.5% (n = 16,020)	290 KD vs. 21,301 non-KD children, Feb 2020–May 2021	[14]
2022/India	COVID-19	Hospitalization: 46.6% ICU admission: 20.4% Mortality: 3.4%	88 CKD children, April 2020–June 2021	[59]
2022/India	COVID-19	Severe disease 22.7% (n = 10) Mortality 4.5% (n = 2)	44 NS children, April 2020–June 2021	[60]
2022/Italy	COVID-19	Morbidity and mortality comparable to general population	43 NS children, 11 studies (review, August 2021)	[61]
2021/Iran	COVID-19	Morbidity: 20.2% (n = 13)	6610 hospitalized children, 65 KD children	[62]
2023/ Mexico	COVID-19	Morbidity: 36% (n = 237) Mortality: 7.6% (n = 50)	366,542 cases < 18 (nationwide), 657 CKD up to July 2022	[19]
2022/ Scotland	COVID-19	Positivity 29.5% (n = 26), Hospitalization:19.2% (n = 5), Hospitalization HR: 11.34 (95% CI 4.6–27.8)	146,183 cases, 5–17 yrs 88 CKD	[18]
2025/ Germany	COVID-19	Mechanical ventilation in acute or chronic kidney failure OR 9.5 [95% CI 4.0–22.2]	3360 cases, COVID-19 hospitalizations	[63]

ESRD: end-stage kidney disease, KT: kidney transplant, HR: hazard ratio, KD: kidney disease, IPD: invasive pneumococcal disease.

**Table 3:** Vaccination rates in children with CKD.

Study Year /Country	Vaccine	Vaccination Rate	Population	Ref.
2010/Greece	H1N1 pandemic influenza	KT: 57.1%, ESRD (PD): 61.4%, CKD: 36.4%, GN (immunosuppression): 26.7%	64 pediatric chronic renal patients	[64]
2024/Turkey	COVID-19	22.9% (n = 11)	48 infected among 220 children with CKD, 2020–2021	[65]
2021/ Europe	PCV	HD: 32.9% PD: 43.9%	357 ESRD children on HD/PD, 16 centers, 11 countries, 2014–1015	[66]
	Influenza	HD: 46.1% PD: 42.4%		
2018/Europe	All recommended vaccines	HBV: 88.6%, PCV/PPSV23: 42%, MMR: 84.9%, VZV: 58.9%, HPV: 27.3% Fully vaccinated: 8.7%	254 ESRD children pre-KT, 4 countries, 16 centers, 1999–2013	[54]
2016/USA	PPSV23	KD: 70.6% NS: 76.6%	102 children 2–21 yrs with KD, including 41 with NS, July 2013–Jan 2014	[67]
	Influenza	KD: 40.2% NS: 66.6%		
2008/Brazil	All recommended vaccines	HBV: 63%, PCV: 10.8%, Measles: 93.5%, VZV: 10.8%, BCG: 100% Fully vaccinated: 19.5%	46 KT children, pre- and post-KT evaluation, October 2001–May 2002	[68]
2017/Israel	Influenza	KT: 84%, NS: 50%, Dialysis: 75%, Total: 45.6%	217 KD children, visiting renal unit, August–October 2011 and September–October 2012	[55]
2014/USA	PCV	0–9 yrs: 10% 15–19 yrs: 20%	515 children with CKD 0–9 yrs, 1528 children with CKD 15–19 yrs, nationwide renal data system report, 2008–2011	[69]
2023/USA	Influenza	Vaccination per season: 0.5–10 yrs: 39% 11–18 yrs:24%	18,203 children and adults < 65 y with glomerular diseases, 2010–2019	[70]

HD: hemodialysis, GN: glomerulonephritis, PD: peritoneal dialysis, NS: nephrotic syndrome, PCV: pneumococcal conjugated vaccine.

**Table 4:** Vaccination recommendations for children with CKD and nephrotic syndrome.

Vaccines Particularly Important for Children with CKD				
Vaccine	CKD-Early Stages	ESRD/Dialysis	Immunosuppression Transplantation	Nephrotic Syndrome
HBV	Routine	Routine/increase dose regimen (country dependent) Revaccination if anti-HBs < 10 IU/L Annual anti-HBs titers booster dose if <10 IU/L	Anti-HBs titers and booster consideration 12 mo post- KT	Routine
PCV/PPSV	Routine	PCV20 booster once, if other than PCV20 vaccine was used for routine immunization PPSV no longer required, for already recipients PCV20 once after 5 yrs		
MMR	Routine	Routine	Officially contraindicated Considered in special circumstances for patients on minimal immunosuppression or minimal dose of steroids (<0.5 mg/kg/d)	
VZV	Routine	Routine		
Inactivated Influenza	Annually	Annually	Annually 3–6 mo post-KT	Annually
COVID-19	Annually	Annually	Annually 3–6 mo post-KT	Annually
HPV	Routine	Routine	Routine 12 mo post KT	Routine
**Other recommended vaccines for children with CKD**				
DTaP/Tdap	Routine	Routine	Routine if not completed pre-KT	Routine
Meningococcal	Routine	Routine	Routine Additional doses might be required for children on anti-C5 agent	Routine
BCG	As per country’s vaccination recommendation CKD/KT not specific indications for administration			

CKD: chronic kidney disease, KT: kidney transplant, MMR: measles, mumps, rubella vaccine, VZV: Varicella Zoster Virus vaccine, DTaP/Tdap: diphtheria, tetanus, pertussis vaccine.

**Table 5:** Barriers to vaccination of children with CKD and interventions for improvement.

Vaccination Barriers	Interventions for Improvement
Vaccine distribution	**Health care system organization:** Vaccine centers for CKD children, ensure sufficiency and accessibility, definition of vaccination teams
Unfamiliarity with guidelines (physicians, families)	**Physicians’ awareness:** education of specialists on vaccination priorities
Confusion of responsibilities (community physicians/specialists)	**Health care system organization:** Targeted nurse-led interventions and EHR system reminders
Live-vaccine safety concerns	**Targeted medical research**
Vaccine hesitancy	**Cocooning strategies**

CKD: chronic kidney disease, EHR: electronic health records.

## Data Availability

Data available upon request.

## Abbreviations

The following abbreviations are used in this manuscript:

CKD	Chronic Kidney Disease
CAKUT	Congenital Anomalies of the Kidney and Urinary Tract
DTaP	Diphtheria-tetanus-acellular pertussis vaccine
DMARDS	Disease-Modifying Antirheumatic Drugs
ESRD	End-Stage Renal disease
FGF	Fibroblast Growing Factor
HD	Hemodialysis
KDIGO	Kidney Disease Improving Global Outcomes
KT	Kidney Transplant
LPS	Lipopolysacharide (Element of Gram—bacteria)
MMR	Measles-Mumps-Rubella
MBL	Manose- Binding Lectin
NHS	National Health System (British guidelines)
NS	Nephrotic Syndrome
GFR	Glomerular filtration rate
IPD	Invasive Pneumococcal Disease
PD	Peritoneal Dialysis
PPV	Pneumococcal Polysaccharide Vaccine (commonly PPV23, protects against 23 types of *Streptococcus pneumoniae*)
PCV	Pneumococcal Conjugate Vaccine (e.g., PCV13, PCV15, PCV20
RAS	Renin-Angiotensin System
SIDKD	Secondary Immunodeficiency related to kidney disease
Treg	Regulatory T cells
